# An Intervention to Increase Outdoor Play in Early Childhood Education Centers (PROmoting Early Childhood Outside): Protocol for a Pilot Wait-list Control Cluster Randomized Trial

**DOI:** 10.2196/38365

**Published:** 2022-07-12

**Authors:** Rachel Ramsden, Christina S Han, Dawn Mount, Janet Loebach, Adina Cox, Susan Herrington, Anita Bundy, Amber Fyfe-Johnson, Ellen Beate Hansen Sandseter, Michelle Stone, Mark S Tremblay, Mariana Brussoni

**Affiliations:** 1 Department of Pediatrics University of British Columbia Vancouver, BC Canada; 2 Department of Design + Environmental Analysis Cornell University Ithaca, NY United States; 3 Department of Landscape Architecture Iowa State University College of Design Ames, IA United States; 4 School of Architecture and Landscape Architecture University of British Columbia Vancouver, BC Canada; 5 Department of Occupational Therapy Colorado State University College of Health and Human Sciences Fort Collins, CO United States; 6 Institute for Research and Education to Advance Community Health (IREACH), Elson S Floyd College of Medicine Washington State University Seattle, WA United States; 7 Department of Physical Activity and Health Queen Maud University College of Early Childhood Education Trondheim Norway; 8 School of Health and Human Performance Dalhousie University Halifax, NS Canada; 9 Healthy Populations Institute Dalhousie University Halifax, NS Canada; 10 Healthy Active Living and Obesity Research Group Children's Hospital of Eastern Ontario Research Institute Ottawa, ON Canada; 11 Human Early Learning Partnership, School of Population and Public Health University of British Columbia Vancouver, BC Canada; 12 British Columbia Injury Research & Prevention Unit British Columbia Children's Hospital Vancouver, BC Canada

**Keywords:** early childhood education and care, preschool, randomized controlled trial, RCT, intervention studies, outdoor play, built environment, pedagogy, behavior mapping

## Abstract

**Background:**

Participation in outdoor play has been extensively documented as beneficial for the health, well-being, and development of children. Canadian early childhood education centers (ECECs) are important settings in young children’s lives and provide opportunities to participate in outdoor play. However, there are barriers to the provision of outdoor play opportunities at ECECs, such as adverse weather conditions, poorly designed outdoor spaces, outdoor time policies, and early childhood educator comfort levels.

**Objective:**

The PROmoting Early Childhood Outside (PRO-ECO) study is a wait-list control cluster randomized trial that evaluates the impact of the PRO-ECO intervention, an innovative outdoor play intervention, on children’s outdoor play behavior. The purpose of this paper was to provide a detailed overview of the pilot study protocol and the methods that will be used to develop, implement, and evaluate the PRO-ECO intervention.

**Methods:**

A total of 8 ECECs delivering licensed care to children aged 2.5 to 6 years in the Greater Vancouver region of British Columbia, Canada, and operated by the YMCA of Greater Vancouver (YMCA GV) are included in this study. Using a wait-list control cluster randomized trial design, we randomly allocated ECECs to either the PRO-ECO intervention arm (n=4) or the wait-list control arm (n=4). The primary outcome measures include changes in the proportion and diversity of observed outdoor play behavior during dedicated outdoor times at the ECECs as measured through observational behavior mapping. Secondary outcome measures include changes in educator attitudes; quality of ECECs’ outdoor play space; and children’s psychosocial strengths, physical activity levels, and social behaviors. A process evaluation of the acceptability of the PRO-ECO intervention in the 8 YMCA GV ECECs will also be assessed. Outcome data will be collected at baseline, 6-month follow-up, and 12-month follow-up. Mixed effect models will test the effect of the PRO-ECO intervention on quantitative outcomes. Baseline and postintervention data will be included in the analysis, controlling for the cluster design. Qualitative data will support quantitative findings and provide evidence for the acceptability of implementation.

**Results:**

Participant recruitment for this study began in August 2021, and baseline data collection was completed at all 8 ECECs in November 2021. As of April 2022, a total of 130 children have been recruited to participate in this study.

**Conclusions:**

The PRO-ECO pilot study will develop, implement, and evaluate the PRO-ECO intervention within 8 YMCA GV ECECs in the Vancouver region of British Columbia, Canada. The findings of this study will be useful for early childhood educators, ECEC providers, and policy makers to consider means for enhancing outdoor play provision and assessing the sustainability of the intervention in ECEC settings.

**Trial Registration:**

ClinicalTrials.gov NCT05075580; https://clinicaltrials.gov/ct2/show/NCT05073380

**International Registered Report Identifier (IRRID):**

DERR1-10.2196/38365

## Introduction

### Background

Outdoor play is “a form of play that takes place outdoors, where the outdoors is defined as any open-air, wild, natural, or human-made space” [1]. The value of outdoor play for children’s health, well-being, and development has been extensively documented [2-8]. Significant evidence outlines the importance of outdoor play in children’s cognitive, physical, emotional, and social development; health; and overall well-being [9-16]. In addition, spending time outdoors can boost children’s vitamin D levels, spatial awareness, and motor skills while offering opportunities to stimulate physical activity [5,6,17]. Despite these benefits, children in North America are spending less time outdoors because of the changing landscape of neighborhoods, increased time spent on technology, and shifting family lifestyles [8,18-21]. Many children have limited access to outdoor environments or face barriers to accessing opportunities for outdoor play [19].

To address declines in children’s opportunities for outdoor play, it is important to develop strategies and interventions that target the early years (0-6 years). Exposure to unstructured outdoor play experiences at an early age promotes positive self-esteem, attention skills, autonomy, and confidence [5,22], and supports lifelong healthier lifestyles [5]. Early childhood education centers (ECECs) are fundamental environments for the early years in Canada, with approximately 60% of children aged 0 to 5 years attending some form of childcare [23]. ECECs provide children with opportunities for outdoor play in environments that they may not otherwise experience in their homes or communities [24]. The provision of opportunities for outdoor play in ECECs depends on the built environment; early childhood educators’ (ECEs) pedagogical approaches, knowledge, and self-efficacy; the policies that guide the delivery of early childhood education (government and program-specific); and the attitudes of parents and communities. Although outdoor play is an essential component of the pedagogy and facility design in ECEC settings in Canada, there is vast diversity in its provision and practice across programs. Many ECECs struggle to provide high-quality and stimulating outdoor play time and can encounter multiple actual and perceived barriers that span individual, interpersonal, organizational, and societal factors such as limited training in supporting outdoor play, excessive fears related to child safety, and deficiencies in the size of and affordances in the outdoor space [25-27].

The early childhood education landscape in British Columbia (BC) is governed by federal, provincial, and municipal policies. ECECs in BC must adhere to provincial Child Care Licensing Regulations, which are regulated by local health authorities across BC [28]. These regulations enforce a minimum of 6 m^2^ of outdoor play area for each child and a minimum of 60 minutes of outdoor active play per day [28,29]. Outside of these requirements, it is up to the individual ECEC to determine the design and use of their outdoor space within the constraints of other licensing regulations.

Ecological models of health behaviors and child development demonstrate that ECEs’ and children’s behaviors are influenced by individual-level factors (eg, ECE knowledge and children’s attitudes), social factors (coworker support and parent knowledge and attitudes), organizational factors (center policy and support), environmental factors (outdoor space and environmental features), and policy factors (licensing and governing policies) [30-32]. Increasing the capacity of ECECs to support high-quality outdoor play experiences for children requires a complex intervention with multiple components addressing the barriers and challenges of the ECECs’ socioecological environment [33]. A complex intervention contains multiple interacting components, requires intervention participants (in this case, ECEs) to engage in several challenging behaviors, targets multiple organizational levels, requires collecting a range of measures to evaluate the intervention’s diverse effects and potential unintended consequences, and allows for flexibility in tailoring the intervention to local circumstances [34].

Previous studies have evaluated play-based interventions to increase children’s physical activity in ECECs [35,36]. However, there is minimal evidence of appropriate interventions that support children’s participation in outdoor play in ECECs. The PROmoting Early Childhood Outside (PRO-ECO) wait-list control cluster randomized trial aims to evaluate the PRO-ECO pilot intervention, a comprehensive outdoor play intervention for children in ECECs. The aim of this paper was to describe the design and protocol of the PRO-ECO study. To our knowledge, this is the first cluster randomized controlled trial to evaluate a comprehensive outdoor play intervention in Canadian ECECs.

### Study Objectives

The PRO-ECO study is guided by the following objectives: (1) to develop and implement the pilot PRO-ECO intervention with overarching components common to all ECEC intervention sites as well as customizable components that are responsive to the needs of each ECEC, (2) to assess the efficacy of the PRO-ECO intervention in increasing and diversifying outdoor play behavior in children aged 2.5 to 6 years, and (3) to assess the acceptability of the PRO-ECO intervention.

## Methods

The methods outlined in this study are informed by the CONSORT (Consolidated Standards of Reporting Trials) statement for cluster randomized controlled trials [37] and the SPIRIT (Standard Protocol Items: Recommendations for Intervention Trials) statement for clinical trial protocols [38] and based on guidance on the development of complex interventions from the evaluation framework of the Medical Research Council [34].

### Study Design

**Figure 1:**
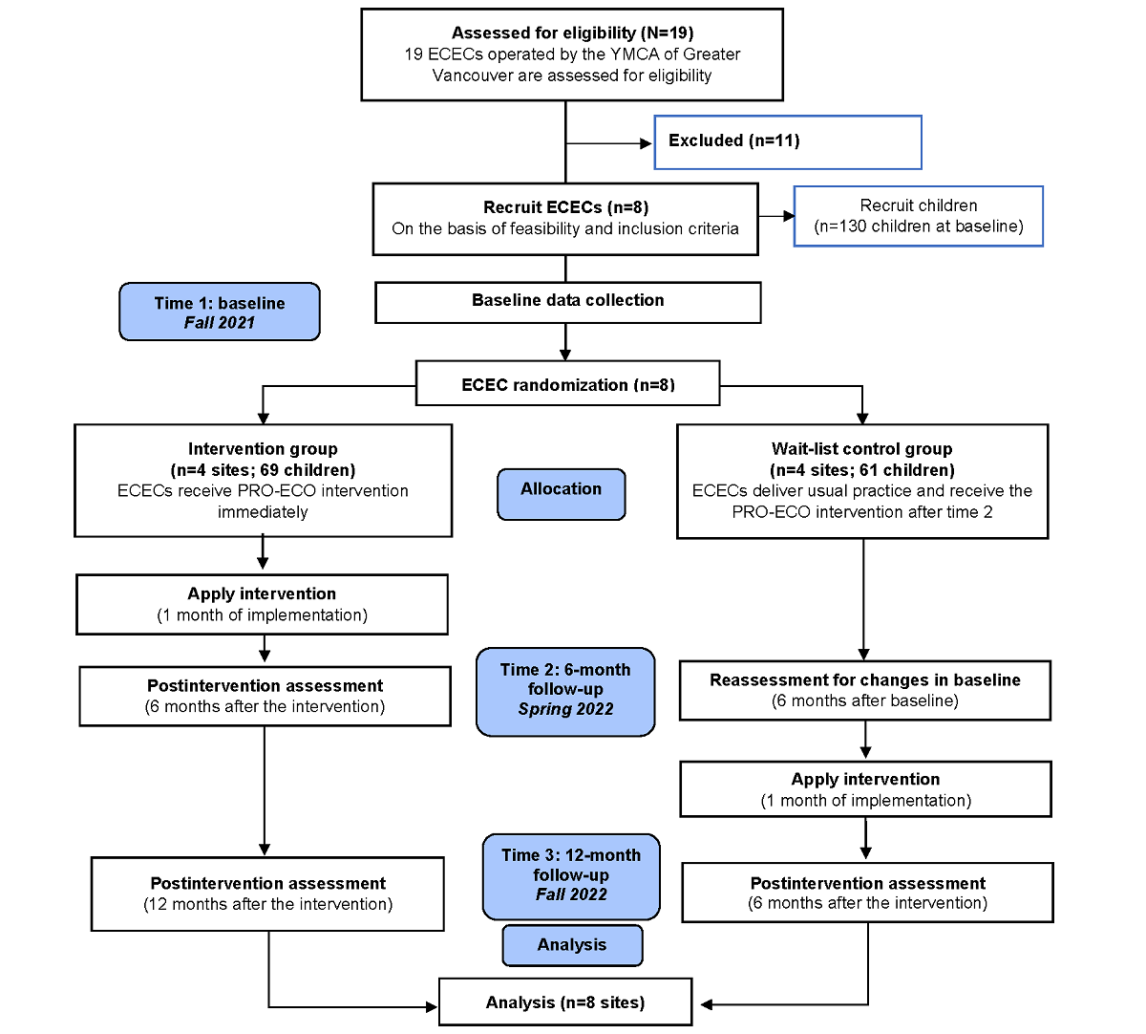
Wait-list control cluster randomized trial flow diagram: PROmoting Early Childhood Outside (PRO-ECO) pilot study. ECEC: early childhood education center.

This study is a pilot wait-list control cluster randomized trial (trial registration NCT05075580) with an intervention arm and a wait-list control arm (Figure 1). A mixed methods approach will be used to collect qualitative and quantitative data designed to meet the study objectives. Quantitative outcomes support the evaluation of the primary outcome variables, and qualitative data assess the acceptability of the implementation of the intervention. Qualitative data will be collected concurrently with quantitative data and allow researchers to explore how the participants experience the intervention.

The study will be conducted in 8 ECECs operated by the YMCA of Greater Vancouver (YMCA GV) from September 2021 to November 2022. Individual ECECs served as the unit of randomization, with 50% (4/8) intervention sites and 50% (4/8) wait-list control sites composing the final sample. Intervention sites received the intervention immediately following baseline data collection, whereas wait-list control sites will receive the intervention 6 months later. Data on outcome measures will be collected at 3 time points: baseline (time 1), 6-month follow-up (time 2), and 12-month follow-up (time 3). Thus, outcome data will assess short- and longer-term outcomes within the intervention group and short-term outcomes within the wait-list control group. Baseline and postintervention data collection will occur during the fall and spring to ensure similar weather patterns at all time points.

### Participant Eligibility and Recruitment

#### ECEC Recruitment

The PRO-ECO intervention will be piloted in 3 cities within the Greater Vancouver region in BC, Canada: Burnaby, Richmond, and Vancouver. The YMCA is one of the largest child care providers in BC, delivering early childhood education services across the province through their local chapters [39]. All 19 ECECs operated by the YMCA GV that provide care for children aged 2.5 to 6 years opt into the Affordable Child Care Benefit, helping ensure they are inclusive of all families. Of these 19 ECECs, 8 (42%) were selected based on their proximity to researchers at the University of British Columbia and their readiness to participate in the study as assessed through informal interviews with YMCA GV staff. Following site selection, each ECEC appointed a staff research lead (*champion*) to liaise between the research team, ECE staff, and parents. In addition, the YMCA GV selected 4 managers to work closely with the research team on all phases of the study. ECEs and ECEC supervisors and managers were also included in this study.

#### Children and Parents

Children were included in this study if they were aged between 2.5 and 6 years, were attending a participating ECEC between September 2021 and November 2021, and had parental consent. Recruitment of children and parents occurred through the champion at each ECEC, who distributed letters and emails and initiated face-to-face conversations. Over the course of the study, participating children may leave their ECEC, and newly enrolled children will be recruited as the study progresses.

#### Sample Size

This study will produce preliminary data for the calculation of a sample size for future studies [40]. Therefore, the sample size for this study is based on the feasibility of the pilot study. Recruitment for this study occurred during the COVID-19 pandemic, and many ECECs were operating with reduced enrollment. A total of 157 children were enrolled in participating ECECs and invited to participate, of whom 82 (52.2%) participated in the intervention arm and 75 (47.8%) participated in the wait-list control arm. Across all 8 ECECs, 82.8% (130/157) of children consented to participate in baseline data collection.

### Randomization, Stratification, and Blinding

Before baseline data collection, we completed a stratified randomization of ECECs based on 2 stratification variables: *percentage of families enrolled in the BC Affordable Child Care Benefit* (<100% or 100%) and *type of facility* (above-grade or at-grade). Information on the type of facility and the percentage of families enrolled in the BC Affordable Child Care Benefit was collected from each ECEC site before randomization. Within each stratum, block randomization was applied to assign each center to the intervention group or the wait-list control group using Research Randomizer [41]. The research trial coordinator (RR) was not blinded to the randomization of each site; however, the research assistants were blinded at baseline data collection. Furthermore, the research staff member performing the data analysis will be blinded to randomization at the data analysis stage.

### PRO-ECO Intervention

#### Intervention Development

Social cognitive theory provides the theoretical base for the PRO-ECO intervention. Furthermore, we are following the intervention mapping approach outlined by Bartholomew et al [42] to ensure that the intervention is grounded in theory, evidence, and the socioecological context and needs of the relevant population. Detailed information on the development of the PRO-ECO intervention can be found in Multimedia Appendix 1 [42-44].

#### Intervention Components

The PRO-ECO intervention is tailored to individual sites. Through the collection of focus group and baseline data, the intervention will be further refined to provide site-specific adjustments, such as specific materials in the built environment design modification or targeted follow-up training and mentorship. The PRO-ECO intervention involves 4 primary components designed to address the complexities and realities of outdoor play participation (Table 1). Funding for the built environment components of the PRO-ECO intervention at each site was provided by the YMCA GV, and the BC Cancer Agency provided monetary funds for the shade-related interventions. In addition to the 4 key intervention components, the study team secured donated rain gear items for children that were distributed to the ECEC if requested.

**Table 1:** PROmoting Early Childhood Outside intervention components.

Intervention component	Intervention activity	Universal vs tailored to ECEC^a^	Socioecological level	Target population
YMCA GV^b^ outdoor play policy	Implementation of organizational “Outdoor Play Policy” across all YMCA GV ECECs that outlines enhanced outdoor play requirements and procedures. A parent handbook outlining outdoor play expectations will also be developed.	Universal	Organization	YMCA GV, ECECs, and ECEs^c^
ECE training	A series of training sessions and opportunities for YMCA ECEs, including: 1-day training that includes content on the importance of outdoor risky play, methods for risk-benefit assessment, and encouragement of the use of loose parts Web-based supplemental training on pedagogical narration ECE outdoor play web-based training tool [45]	Universal	Individual and center	ECE
ECE training	Ongoing monthly and as-needed supportive training and mentorship provided by YMCA senior managers and research team	Tailored	Individual and center	ECE
ECEC outdoor space modification	Each ECEC site will undergo an outdoor space modification as follows: Design plans for each center will be based on the Seven Cs and developed by 14 University of British Columbia School of Architecture and Landscape Architecture graduate students in a design studio. Graduate students will co-design modifications to the built environment with ECECs. Graduate students will implement modifications. A budget of CAD $4000 (US $3181.36) for general expenses and CAD $2000 (US $1590.68) for shade-related interventions is available for each site.	Tailored	Center	ECEC, children, and ECEs
Parent engagement	Parent engagement events will be provided to increase knowledge of the importance of outdoor play and encourage parent involvement in implementing the outdoor space modification. Pedagogical narration of children’s outdoor play experiences and learning will be posted by ECEs on the internal YMCA mobile app for access by parents.	Tailored	Center	Parents and community

^a^ECEC: early childhood education center.

^b^YMCA GV: YMCA of Greater Vancouver.

^c^ECE: early childhood educator.

#### Delivery of the Intervention

The intervention is split into 2 phases: the introduction phase and the maintenance phase. The introduction phase includes the implementation of the PRO-ECO intervention at the 4 intervention sites and takes up to 1 month. The maintenance phase is in place for 11 months after the PRO-ECO intervention is completely implemented in the intervention group and for 5 months for the wait-list control group. It involves maintaining the components of the intervention throughout the course of the study. The maintenance phase ends once the postintervention data are collected at time 3 (fall 2022).

#### Intervention Group

ECECs randomly assigned to the intervention group received the PRO-ECO intervention immediately following completion of time 1 baseline data collection. At time 2 in spring 2022, 6-month postintervention data will be collected. At time 3 in fall 2022, 12-month postintervention data will be collected.

#### Wait-list Control Group

ECECs randomly assigned to the wait-list control group continued with their normal daily practice, including standard curriculum and outdoor play time after time 1 baseline data collection. At time 2 in spring 2022, additional baseline data will be collected. The 4 wait-list control ECECs will receive the PRO-ECO intervention after time 2 data collection has occurred. Time 3 postintervention data collection will be completed 6 months after receiving the intervention, in fall 2022.

### Data Collection and Measures

#### Primary Outcomes

The primary trial outcome is the occurrence of outdoor play behavior at ECECs during designated outdoor play times. Play behavior is coded using the expanded version of the Tool for Observing Play Outdoors (TOPO) developed by Loebach and Cox [46] and captured using a systematic observational mapping protocol. The TOPO measures children’s play behavior through validated categories of 8 play types and 1 nonplay type along with their corresponding subtypes (Table 2). For this study, we will code play behavior and nonplay behavior at the subtype level at each ECEC, with all play types being categorized as *Play* and all nonplay types being categorized as *Nonplay* for the analysis of our primary outcome. In addition, diversity of children’s outdoor play behavior will be examined using proportions of different play types.

The TOPO is implemented using a place-based observational behavior mapping (OBM) protocol. OBM strives to understand how an environment supports movement and use behaviors by mapping, recording, organizing, displaying, and analyzing geographically located data [47,48]. Base maps are used to provide an overview of the given environment, and predetermined observable data variables are collected. Each ECEC outdoor play space was divided into 2 measurement zones. In total, 2 researchers (one in each zone) will conduct observations at each ECEC as children participate in designated outdoor play time as determined by the ECEC. Researchers will scan each zone in a counterclockwise direction selecting the first child to enter the viewpoint. The researchers will then capture a 15-second video of the child’s outdoor play and assign play behavior data to the primary activity performed during the video. A total of 200 fifteen-second play events will be compiled and coded for each ECEC site at each data collection time point (time 1, time 2, and time 3). The observational data will be collected using a place-centered approach that captures play behaviors of a range of children across the *space* of the ECEC rather than a person-centered approach that focuses on individual children. During each observation period, if there is no child in a given observational zone, a note will be made to indicate that no child was playing in that zone at that time. Additional variables will be collected through OBM and are outlined in Textbox 1.

The reliability of the OBM method is defined by the degree of interrater reliability and agreement, which will be assessed using weighted κ and intraclass correlation coefficients [49,50]. All researchers will participate in training sessions on the OBM methodology, and interrater reliability and agreement will be assessed at this time. In addition, a 10% sample of data at each time point will be recorded to assess the interrater reliability and agreement. A κ value of ≥0.70 will be used as commonly accepted as adequate for scientific research [49].

**Table 2:** Tool for Observing Play Outdoors developed by Loebach and Cox [46].

Play type and subtype			Description
**Physical play**			
	Gross motor	Using large muscles, whole body movement, large muscle activities that require hand-eye coordination	
	Fine motor	Smaller muscle movements and hand-eye coordination, picking up or manipulating small objects	
	Vestibular	Activities that test and improve sense of balance or reinforce their relationship to the earth, movement of the head or quick movements in multiple directions	
	Rough and tumble	Engagement in playful or mock fighting or wrestling or more broadly playful physical contact	
**Exploratory play**			
	Sensory	Primarily passive (ie, nonmanipulative) exploration of an object or environment, focused sensory attention	
	Active	Active manipulation of an object or the environment	
	Constructive	Physically building or constructing something or thoughtful destruction or taking apart of something	
**Imaginative play**			
	Symbolic	Using an object, action, or idea as a symbol for something else with no evidence of sociodramatic or fantasy	
	Sociodramatic	Pretending typical social, domestic, or interpersonal experiences or roles they may experience as adults	
	Fantasy	Enacting something that is unlikely to occur in real life	
**Play with rules**			
	Organic	2 or more kids agree to play or challenge each other in a certain way where they develop, negotiate, or change the rules as they go	
	Conventional	2 or more kids play games that have common, universal, or well-known rules that the players understand	
**Bio play**			
	Plants	Observes, discusses, or interacts with a living plant or parts of the plant (flowers or seed pods)	
	Wildlife	Observes, discusses, or interacts with wildlife (that is not a domestic pet)	
	Care	Acts in a way that demonstrates care or stewardship for the environment or an appreciation of nature	
**Expressive play**			
	Performance	Intentionally performing for others in some way	
	Artistic	Manipulating the environment specifically for an artistic, creative, or esthetic outcome	
	Language	Activities involving the playful use or testing of sound, words, or language	
	Conversation	Primary interaction is social conversation with children or adults	
**Restorative play**			
	Resting	Taking a mental break or rest	
	Retreat	Remove themselves to a small, controlled space, may watch others	
	Reading	Reading or writing for pleasure or listening to others or music	
	Onlooking	Child deliberately steps back from nearby play for a period of observation	
**Digital play**			
	Device	Playing with or on a digital device with no interaction with the environment	
	Augmented	Using a digital device to augment their interaction with the physical world	
	Embedded	Interacting with digital prompts or devices embedded in the environment without a personal digital device	
**Nonplay**			
	Self-care	Taking care of themselves or their appearance, can include helping another with these activities	
	Nutrition	When a child is taking a break to eat or drink	
	Distress	When a child is disengaged from play and exhibiting signs of distress	
	Aggression	Refers to nonplayful, antagonistic interactions with another child or adult	
	Transition	Nonplayful movement from one space to another, no active engagement or exploration of the environment	
	Other	Other types of observed “nonplay” activities, can include “chores” or cleanup work

**Collected variables within the observational behavior mapping protocol.**

**Variables and levels**

- Sex: male, female, and unknown
- Play type (see Table 2 for subtypes): physical, imaginative, bio, restorative, exploratory, play with rules, expressive, digital, and nonplay
- Risk-taking behavior: risk avoidance, exploratory risk appraisal, low or no risk, low-risk positive, low-risk negative, moderate-risk negative, moderate-risk positive, high-risk negative, and high-risk positive
- Play communication: play, environment, peer-social, adult-social, cowabunga!, self-talk, could not hear, ask for help, none, and other
- Adult interaction: no adult present, observing, participating, directing, restricting, and other
- Physical activity intensity: stationary or motionless, stationary with limb or trunk movements, slow or easy movement, moderate movement, and fast movement
- Peer interaction: solitary, parallel, cooperative, onlooking, unoccupied, and conflict
- Group size: open text
- Environmental interaction: fixed manufactured, fixed natural, loose manufactured, loose natural, and narrative text
- Interacted with coder: yes and no
- Adult says *Be careful*: yes and no
- Open coding: open text

#### Additional Outcome Measures

##### Attitudes Toward Outdoor Risky Play

The effect of the intervention on ECEs’ tolerance of risk in play will be assessed using the Teacher Tolerance of Risk in Play Scale [51]. The Teacher Tolerance of Risk in Play Scale is a 25-item instrument that has been validated for use as a measure of intervention effects aimed at increasing children’s access to risky play (a fundamental component of outdoor play) [51]. This measure will be administered to all ECEs at the 8 study sites during all data collection phases (times 1, 2, and 3).

##### Quality of ECEC Outdoor Spaces

The Seven Cs framework will form the basis for the assessment of outdoor space quality at all ECEC sites and the reassessment of the environment at ECEC sites after implementation of the PRO-ECO intervention and will guide the development of the plan for modification of the outdoor environment [52]. The Seven Cs framework was designed to provide guidance in the design of outdoor play spaces for children in early childhood settings based on 7 criteria: character, context, connectivity, change, chance, clarity, and challenge [52]. The Seven Cs assessment tool for ECECs will be used for baseline and postintervention measurement [53].

##### Acceptability of the PRO-ECO Intervention

As this study pilots an outdoor play intervention that can be replicated at other ECECs, it is imperative to assess the process of intervention implementation and acceptability (from the perspective of ECEs, parents, and YMCA GV managers). In this study, acceptability relates to the willingness of individuals (ECEs, parents, and children) and the organization (YMCA GV) to participate in the intervention, inform future recommendations, and apply the intervention for future use. Qualitative data will be collected before and after the intervention to understand the acceptability of the PRO-ECO intervention by the target populations at each site. Focus groups and individual key informant interviews will be organized with ECEs and ECEC administrators, who can provide critical and reflective information about the acceptability of the intervention. To understand parents’ perceptions of the acceptability of the PRO-ECO intervention, a purposive sample of parents will be engaged to participate in intercept interviews. The intercept interview method provides a convenient way of interviewing the target population at the time and location most relevant and convenient in the context of the study [54]. In our study, it will be at the time of child drop-off or pick-up at their ECEC. Intercept interviews will be approximately 10 minutes long to accommodate parents’ busy schedules.

Semistructured interview guides have been developed for the focus groups, key informant interviews, and intercept interviews with parents. These interview guides were developed by theorizing the constructs of acceptability in our study [55], such as perceived change in children’s outdoor play because of the intervention, feasibility for broad implementation, and the cost-benefit of the intervention. Focus groups will be administered to discuss children’s outdoor play, the challenges they are experiencing, and their suggestions for change, including modifications to the outdoor space. Qualitative methods will be administered at time 1 (baseline) for all sites, at time 2 for intervention ECECs, and at time 3 for wait-list control ECECs.

##### Economic Evaluation of the PRO-ECO Intervention

An economic evaluation will examine the costs of implementation of the intervention and the cost versus benefits of the PRO-ECO intervention. YMCA GV administrative data that can be monetized will be collected to consider the monetary benefits of the PRO-ECO intervention in comparison with the capital costs of intervention administration. Information will be collected throughout the study (Textbox 2) to compile a comprehensive economic evaluation guided by the framework by Levin and Schwartz [56].

An additional economic analysis will also consider the resources and costs associated with expanding the PRO-ECO intervention to future ECEC sites.

**Information collected throughout the study to compile a comprehensive economic evaluation.**

**Information for economic evaluation**

- Ongoing administrative costs specific to the PROmoting Early Childhood Outside (PRO-ECO) intervention in both the introduction and maintenance phases (ie, beyond usual early childhood education center [ECEC] programming costs):
  Capital costs of using, maintaining, and staffing ECECs
  Out-of-pocket ECEC expenses
  Early childhood educator (ECE) and manager time commitment
  ECE sick days
  ECEC staff turnovers
  Children’s attendance rates
  Reported incidents of challenging child behaviors
  Reported incidents of child injuries
  Benefits of the PRO-ECO intervention (outdoor play proportion)
- Implementation costs of the PRO-ECO intervention components:
  ECE training (eg, total hours spent training and average hourly pay of trainers)
  Built environment modifications (eg, supply costs, total hours spent purchasing supplies, total hours spent designing built environment modifications, total hours spent modifying the built environment, and average hourly pay of all parties involved)
  Parent engagement (eg, total hours spent engaging with parents and average hourly pay of trainers)
  Additional outdoor gear for children and ECEs (eg, jackets, boots, and rain ponchos)

##### Child Health, Development, and Well-being

The psychosocial strengths of children will be assessed using the Strengths and Difficulties Questionnaire, teacher version [57], which includes 25 items across 5 scales measuring emotional symptoms, conduct problems, hyperactivity or inattention, peer relationship problems, and prosocial behavior. When combining the subscales without the prosocial scale, a total difficulty score is provided to outline psychosocial challenges and strengths [58]. In addition, ECEs’ perceptions of children’s confidence, motivation, knowledge, and understanding of outdoor play participation will be captured through ECEs’ pedagogical narratives, focus groups, and interviews. Children’s physical activity intensity will be measured using the Children’s Activity Rating Scale (CARS) as part of the OBM protocol [59]. Injury incidents will be measured through the abstraction of data from ECEC incident report forms. The Preschool Social Behavior Scale-Teacher Form [60] will be used to measure child development and behavior outcomes, including 19 items measuring relational aggression, overt aggression, prosocial behavior, and depressed affect. Child health, development, and well-being outcomes will be assessed at baseline (time 1), 6-month follow-up (time 2), and 12-month follow-up (time 3).

#### Study Covariates

Covariates for this study will include demographic information of children, including sex, age, first language spoken at home, family composition, average household income, and the highest level of education completed by a household member. The demographic information of the children will be collected using parent-reported questionnaires. In addition, the child’s length of time at the ECEC and other forms of formal care, as well as the type of care (full-time or part-time), will be collected. At the time of data collection, data on the weather, temperature, and time of day will be recorded. The study covariates will be collected at each data collection time point (time 1, time 2, and time 3).

The following information related to recruitment, retention, and attendance will be collected throughout the intervention period (1 year) for both the intervention and control sites: (1) number of eligible children that were approached to participate in the study; (2) number of children who consented to participate, did not consent to participate, or did not respond; (3) number of children who withdrew from the study; (4) number of children who enrolled in the ECECs after initiation of the intervention; (5) number of children who left the ECEC after initiation of the intervention; and (6) individual and center-wide attendance rates at each ECEC. An overview of outcome variables measured as part of the PRO-ECO study is shown in Table 3.

**Table 3:** Outcome variables and measures for the PROmoting Early Childhood Outside (PRO-ECO) study^a^.

Outcome, subcategory, and variable				Measure		Informant						
						Child		ECE^b^		ECEC^c^		Parent
**Primary outcomes—children**												
	Occurrence of outdoor play		TOPO^d^—play type		✓							
	Diversity of outdoor play behavior		TOPO—play type		✓							
**Additional outcomes**												
	**ECEC**											
		Quality of ECECs’ outdoor play space	Seven Cs ECEC assessment score						✓			
		ECEs’ attitudes toward risky play	T-TRiPS^e^				✓					
		Acceptability of PRO-ECO intervention	Interviews and focus groups with ECEs and administrators; intercept interviews with parents				✓				✓	
		Economic analysis of PRO-ECO intervention	ECE and child attendance, ECE staff turnover, incidents of children’s challenging behaviors, institution and intervention costs, and nonfinancial outcomes (outdoor play)				✓		✓			
	**Child health, development, and well-being**											
		Psychosocial	SDQ^f^		✓							
		Injury	Reported incidents						✓			
		Physical activity intensity	OBM^g^ (CARS^h^)		✓							
		Development and behavior outcomes	PSBS-T^i^				✓					

^a^All variables will be collected at each time point (time 1, time 2, and time 3).

^b^ECE: early childhood educator.

^c^ECEC: early childhood education center.

^d^TOPO: Tool for Observing Play Outdoors.

^e^T-TRiPS: Teacher Tolerance of Risk in Play Scale.

^f^SDQ: Strengths and Difficulties Questionnaire.

^g^OBM: observational behavior mapping.

^h^CARS: Children’s Activity Rating Scale.

^i^PSBS-T: Preschool Social Behavior Scale-Teacher Form.

### Analysis

#### Primary Outcome Analysis

The proportion of play occurrence in comparison with nonplay occurrence across ECECs at baseline will be summarized by intervention group using frequency and percentages. This follows a similar analysis completed by van Dijk-Wesselius et al [61], who used a comparison of children’s play and nonplay behavior before and after the intervention as an effective and significant measurement outcome of children’s outdoor play occurrences. The frequencies and percentages of all play types will also be summarized by treatment group to provide a descriptive overview of diversity of play. This follows previous descriptive analyses completed by Loebach et al [48,62], who used OBM data to examine the frequency and diversity of play types. Baseline demographic and ECEC characteristics will be summarized by group using mean and SD and median and IQR for continuous variables, and frequencies and percentages for categorical characteristics. Bivariable relationships between children’s demographic characteristics and outdoor play occurrence, as well as by intervention group, will be explored to assess for potential confounding at an individual level given that randomization is at the cluster level. Mixed effect models will be used to assess differences in quantitative outcome measures between the intervention and wait-list control groups as well as within-group comparisons of pre- and postintervention measures.

#### Cost-benefit Analysis

Cost-benefit analyses aim to estimate the costs and benefits of a particular policy or program and determine whether the societal impacts are worth the investment. For both short- and long-term cost-benefit analyses, we will (1) estimate the economic values of the costs and benefits for the a priori variables of interest (capital costs and changes in occurrence of outdoor play) and (2) apply an investment criterion to the estimated values of costs and benefits [63]. Our analysis framework will be based on decades of published methods from economists evaluating the Perry Preschool project [64-67], a longitudinal study that followed preschool-aged children from disadvantaged backgrounds through adult life [63,68]. To assess societal effects, we will use a net present value criterion to account for benefits and costs that vary over time [63]. For the cost-effectiveness analysis, we will examine differences in outdoor play occurrences per dollar spent on the PRO-ECO intervention versus traditional outdoor play delivery [69].

#### Qualitative Analysis

Qualitative interview data from ECE focus groups, individual key informant interviews, and intercept interviews with parents will be analyzed using the qualitative content analysis method [70]. Theorized constructs of acceptability, which inform our interview guides, will also be used as the analytical framework to understand and categorize participant narratives. Given that the primary aim of the qualitative interviews is to describe participants’ acceptability of the intervention, this deductive analytical approach will serve well in providing focus and content for different constructs of our interest. New concepts will also be constructed that are not fully captured or described by the existing analytical framework, and concepts that are deemed necessary for further exploration will be analyzed anew using the thematic analysis method [71]. More specifically, we will follow 6 phases of reflexive thematic analysis—*familiarization, generating initial codes, searching for themes, reviewing themes, defining and naming themes, and producing the reports*—to go beyond descriptive reports and yield a more complex and nuanced account of a phenomenon or experience.

### Ethics Approval

Ethics certification was received from the University of British Columbia and the Children’s and Women’s Health Centre of British Columbia Research Ethics Board (H20-03912).

## Results

This study was developed to implement and evaluate the PRO-ECO intervention. Funding to conduct this study was confirmed in January 2021. Ethics approval through the University of British Columbia and the Children’s and Women’s Health Centre of British Columbia Research Ethics Board was received in March 2021, and participants were recruited beginning in August 2021. Baseline data were collected from October 2021 to November 2021, and the intervention ECEC sites received the PRO-ECO intervention in December 2021. As of April 2022, a total of 130 children have been recruited to participate in this study.

## Discussion

### Overview

This study is novel in building and evaluating a comprehensive intervention to enhance outdoor play in Canadian ECECs. The PRO-ECO intervention addresses ECECs’ socioecological context, including the ECEs’ individual knowledge, attitudes, and behaviors; parents’ knowledge and attitudes toward outdoor play; the quality of the outdoor play space; and the policies governing the facility. The intervention is underpinned by social cognitive theory using evidence-based behavior change techniques to foster change. It includes aspects that can be universally applied but also the flexibility to tailor to local needs and context. Using a mixed methods, wait-list control cluster randomized trial design, the implementation and efficacy of the PRO-ECO intervention can be assessed.

The results and lessons learned through this study will inform the feasibility of a full-scale randomized trial that continues to assess the effectiveness of the intervention as well as help develop guidelines for the implementation of the PRO-ECO intervention in other ECECs. Furthermore, the health economic analyses will generate data to inform the sustainability of future academic and health policies in ECECs.

### Strengths and Limitations

A strength of this study and the development of the PRO-ECO intervention is that an interdisciplinary stakeholder committee has been gathered to inform best practices and the primary components of this intervention. The process has included extensive partnership and consultation with YMCA GV management; ECEs; licensing officers; and multidisciplinary experts in early childhood education, landscape architecture, public health, outdoor play, and child development.

The PRO-ECO study will provide evidence-based information on the curriculum, policies, outdoor environments, and professional development training that support outdoor play opportunities among children in ECECs. The results of this study may be applied broadly through the expansion of the intervention to other YMCA ECECs and potentially other child care programs across Canada. This study will also provide insightful alignment with ongoing international research on outdoor play in ECECs, such as a recent study published in Norway [27] and research in Washington on their newly licensed outdoor ECEC programs [72].

In designing the PRO-ECO study, the research team identified common challenges when implementing an outdoor play intervention in ECECs and aimed to address them in the study design. However, because of the complexity of this study design, there are limitations that are anticipated throughout this study. First, the PRO-ECO intervention is a pilot study and, therefore, the sample size is based on feasibility while retaining optimal statistical power. To understand if the PRO-ECO intervention can be administered and assessed in a larger sample size, a sample of 8 ECECs is included in this study. This sample size could limit the identification of our estimated effect size between the intervention and control groups. The site inclusion criteria consider the readiness of the site to participate and the geographic area where the site is located. In addition, stratified randomization strives to create an intervention and control group that are similar while also ensuring there is a diverse representation of child care sites in the Greater Vancouver region. However, although considerations were made to create a diverse and representative sample of child care sites, we cannot account or stratify for all site characteristics.

Although this study strives to measure change in the occurrence of outdoor play before and after the intervention in comparison with nonplay, there is a possibility that the amount of *nonplay* at baseline is already low and would be difficult to reduce further. In this case, it may not be possible to detect a significant increase in the occurrence of outdoor play after the implementation of the intervention. Examining diversity of play as an additional primary outcome measure will provide more information to understand changes in outdoor play frequencies before and after the intervention and inform play measures for future studies. The CARS is a direct observation method used to measure physical activity levels among children and was selected based on the financial resources available for this study. In comparison with the use of indirect calorimetry, accelerometry, and heart rate monitoring to measure children’s physical activity levels, the CARS may be subject to measurement error. However, all researchers collecting these data were extensively trained on the use of this tool, and previous studies have validated the use of direct observation methods, including the CARS, to assess children’s physical activity [73].

Postintervention data collection will occur 1 year after baseline. It is expected that some children will be lost to follow-up as a result of leaving the ECEC or withdrawing consent. As indicated earlier, primary variable data will be collected at the center level, allowing us to study ECECs rather than individual children. Although the children participating in this study may change, we will engage additional children for postintervention data collection as needed. We anticipate study contamination between sites as ECEs can be moved between sites to accommodate staffing shortages. Furthermore, the champions at each site attend a weekly PRO-ECO project meeting where logistics are discussed and would be aware of the general activities involved in implementing the PRO-ECO intervention.

In addition, the design of this study commenced in 2019, before the COVID-19 global pandemic. Although ECECs continue to function and provide essential care to many families, we recognize that this has caused changes in the delivery of education and care across Canada. Disruption related to enrollment rates, staffing, or outdoor play practices at ECECs may cause limitations to participation and data collection in our study.

## Appendix

Multimedia Appendix 1 PROmoting Early Childhood Outside intervention development.

## Abbreviations

BC: British Columbia
CARS: Children’s Activity Rating Scale
CONSORT: Consolidated Standards of Reporting Trials
ECE: early childhood educator
ECEC: early childhood education center
OBM: observational behavior mapping
PRO-ECO: PROmoting Early Childhood Outside
SPIRIT: Standard Protocol Items: Recommendations for Intervention Trials
TOPO: Tool for Observing Play Outdoors
YMCA GV: YMCA of Greater Vancouver
