# Supplementary material for: An Intervention to Increase Outdoor Play in Early Childhood Education Centers (PROmoting Early Childhood Outside): Protocol for a Pilot Wait-list Control Cluster Randomized Trial
Source: JMIR Res Protoc. 2022 Jul 12;11(7):e38365. doi: 10.2196/38365 (PMC9328786; doi:10.2196/38365)

# Multimedia Appendix 1: PRO-ECO Intervention Development

Theory-based interventions that use appropriate behaviour change strategies and address multiple components of the environment are more likely to result in behaviour change in ECECs. The PRO-ECO intervention is grounded in social cognitive theory (SCT) [43]. SCT is well suited to considering methods for modifying behaviours that are complex and require considerable capability, such as supporting children’s outdoor play in ECEC environments. According to SCT, determinants of behaviour include outcome expectations (“When I support outdoor play in my ECEC, my relationship with the children will improve”), outcome expectancies (“Fostering children’s joy is something I value”), self-efficacy (“I am confident that I can support children’s risk taking”), behavioural capability (“I know *what* behaviour I need to engage in and *how* to perform that behaviour”), perceived behaviour of others, social environments (colleagues, families), and physical environments (outdoor space). SCT considers that barriers can be challenging to overcome, even when self-efficacy is high [42].

SCT stresses the importance of learning through observation of others modeling the desired behaviour, with four processes that the learner must undertake or experience, including understanding the relevant aspects of the behaviour, retaining the knowledge, producing the action, and feeling motivated. Peers that the learner feels are similar to them are critical for peer behaviour modeling to be effective (i.e., “if they can do it, I can too”). However, it is also necessary to focus on fostering social and physical environments that facilitate change by reducing barriers and/or by making it easier for the learner to undertake the behaviour.

SCT stresses behaviour change methods that are based on active learning with the learner performing the behaviour during the learning process. Behaviour change techniques include peer modeling (seeing that a person they can relate to is capable of accomplishing the behaviour; demonstrating the series of small steps necessary to attaining the larger goal), reinforcement (vicarious, external or internal), enactive mastery experiences (experiencing success in ever-more challenging situations), strong encouragement (repeated verbal persuasion that the person can do it), and supporting positive physical and emotional states (reducing stress while building positive emotions).

The PRO-ECO intervention is based on the principles of SCT, acknowledging and addressing the ECEC’s social and physical environment, as well as working with ECEs to support behaviour change. The PRO-ECO intervention was developed with the support of a comprehensive

Table 1: Intervention mapping approach to PRO-ECO intervention.

| **Step** | **Activity** |
| --- | --- |
| 1.Understanding the problem | Established an advisory group with administrators, leading academics, and licensing officers for consultation on intervention design and study methods.Established a working group that included the Principal Investigator, Research Coordinator and ECEC managers overseeing the study sites. The working group met on a weekly basis throughout project planning. Site champions joined the working group once they were identified. The working group met weekly during project planning and implementation.Conducted a needs assessment of the target population. This involved five focus groups with 40 ECE professionals (staff, administrators, LOs, trainees).Developed a logic model of the problem (Figure 1). |
| 2. Intervention outcomes and objectives | Developed a logic model of change (Figure 2) to identify behaviour and environment change objectives (what needs to change and for whom) and behavioural and environmental outcomes anticipated as a result of the intervention. |
| 3. Intervention design | SCT theory guides the intervention and choice of behaviour change methods.Focus groups with ECEs at each site during baseline data collection informed tailoring of the intervention components.The training components of the intervention were pre-developed.Outdoor risky play full-day workshop and monthly refresher check-ins developed by the YMCA Southwestern Ontario and delivered by local YMCA training staff.Training on pedagogical narration is already standard for all YMCA ECEs. However, the ECEC sites in the intervention group received refresher training that focuses on documenting the learning that occurs in outdoor spaces.Digital self-guided outdoor play training for ECEs available online at OutsidePlay.ca [44].Additional intervention components were co-developed with the YMCA GV and project partners.YMCA outdoor play policyDesign and modification of the outdoor environmentParent engagement strategies |
| 4. Intervention production | Continued consultation with the working group to plan implementation of the intervention.The design modification and parent engagement strategies are adapted and tailored to each ECEC site to reflect the needs of their outdoor environment and parent community. |
| 5. Intervention implementation plan | Intervention implementation as well as launch of the pilot RCT requires extensive engagement with each site.Short video describing the project, its purpose and methods posted on the Brussoni Lab website for access by ECEs and parentsPRO-ECO site handbooks provided to each site with a calendar of study activities, an infographic describing the study, and QR code to study websiteInfographic posters and bookmarks describing the study with QR code to study website for posting and distributing to parents at each site |
| 6. Evaluation plan | Intervention evaluation via a wait-list control cluster RCT |

##### Figure 1: PRO-ECO intervention logic model of the problem

### **
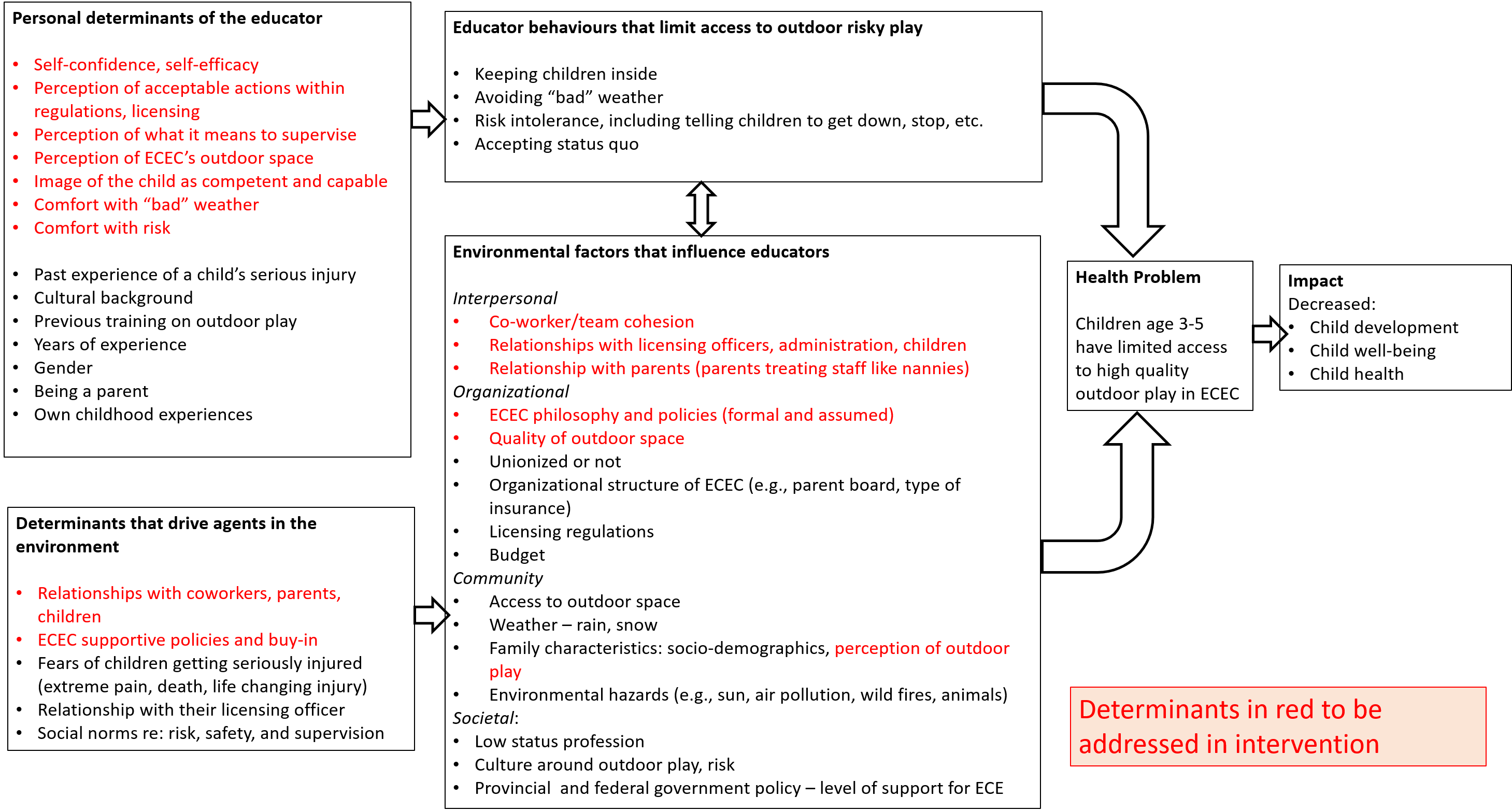
**

##### Figure 2: PRO-ECO intervention logic model of change


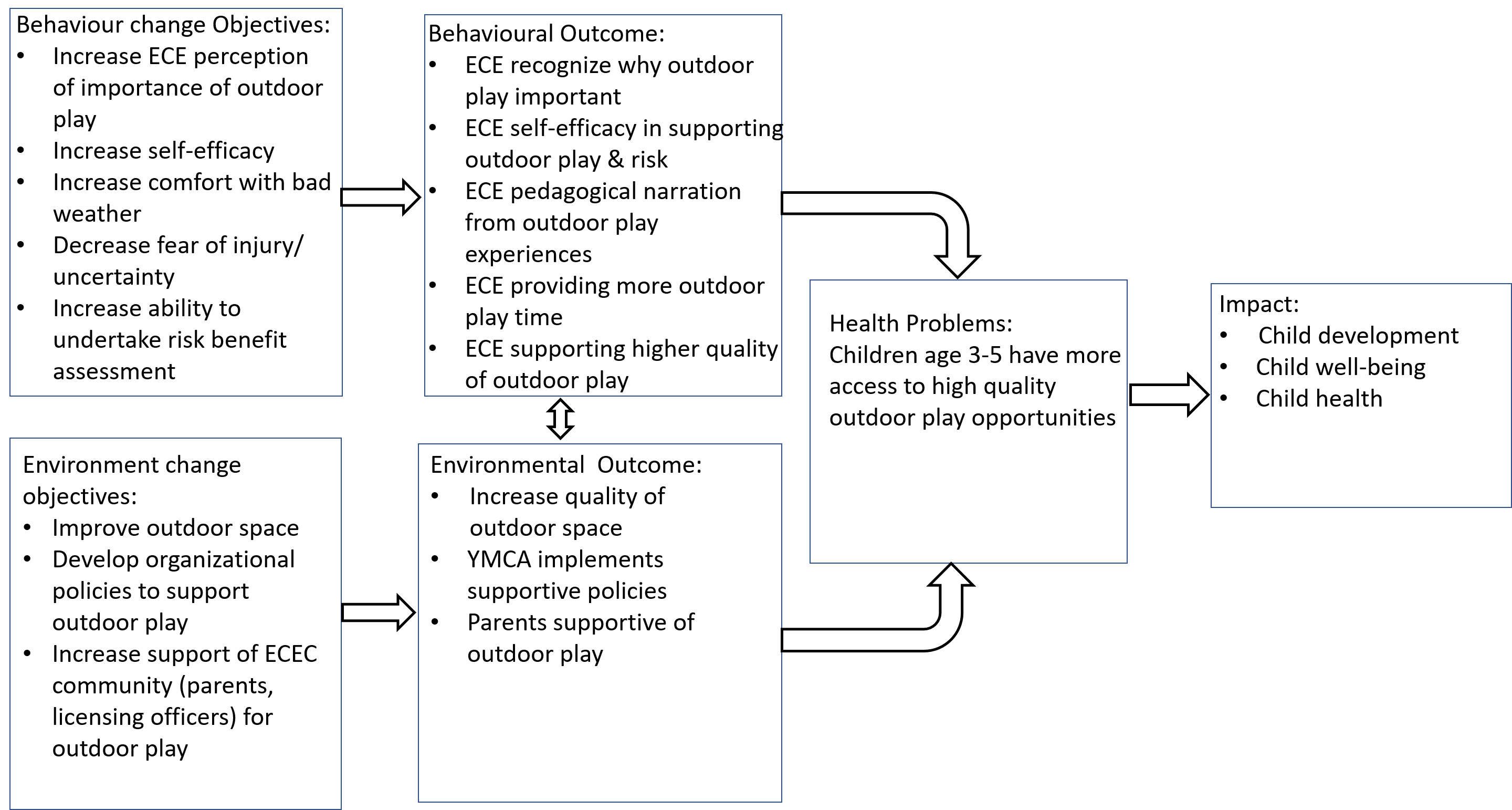

Supplement: Multimedia Appendix 1 [file resprot_v11i7e38365_app1.docx]
